# S100A2 induces epithelial–mesenchymal transition and metastasis in pancreatic cancer by coordinating transforming growth factor β signaling in SMAD4-dependent manner

**DOI:** 10.1038/s41420-023-01661-1

**Published:** 2023-09-27

**Authors:** Qinbo Chen, Hangcheng Guo, Haojie Jiang, Zujian Hu, Xuejia Yang, Ziwei Yuan, Yuanyuan Gao, Ge Zhang, Yongheng Bai

**Affiliations:** 1 Key Laboratory of Diagnosis and Treatment of Severe Hepato-Pancreatic Diseases of Zhejiang Province, The First Affiliated Hospital of Wenzhou Medical University, 325000 Wenzhou, China; 2 Department of Orthopedics, The First Affiliated Hospital of Southwest Medical University, 646000 Luzhou, China; 3 National Key Clinical Specialty (General Surgery), The First Affiliated Hospital of Wenzhou Medical University, 325000 Wenzhou, China

**Keywords:** Metastasis, Oncogenesis

## Abstract

Pancreatic ductal adenocarcinoma (PDAC) is a highly aggressive tumor and is associated with a poor prognosis. Treatment strategies for PDAC are largely ineffective primarily because of delay in its diagnosis and limited efficacy of systematic treatment. S100A2 is associated with the proliferation, migration, and differentiation of several tumors; however, its effects on PDAC and the associated molecular mechanisms remain to be explored. We studied the mechanisms underlying the effect of S100A2 on epithelial–mesenchymal transition (EMT) and metastasis in PDAC cells. We found that the level of S100A2 remarkably increased and was associated with poor PDAC prognosis. The overexpression of S100A2 in PANC-1 cells also induced EMT, in addition to increasing the invasion and migration of PDAC cells, whereas the knockdown of S100A2 markedly inhibited cell metastasis. Furthermore, S100A2 was found to enhance metastatic abilities in vivo. The overexpression of S100A2 increased SMAD4 expression, whereas the knockdown of S100A2 reduced SMAD4 expression. SMAD4 overexpression could effectively rescue the effects of S100A2 knockdown on EMT. S100A2 mechanistically activated the transforming growth factor (TGF)-β/Smad2/3 signaling pathway, upregulated SMAD4 expression, induced EMT, and increased PANC-1 cell metastasis. In conclusion, the S100A2/SMAD4 axis modulates EMT to accelerate PDAC development. Our results supplement and enrich the understanding of the pathogenesis underlying PDAC and provide a new theoretical basis and strategy targeting S100A2 for the diagnosis and treatment of PDAC.

## Introduction

Cancer has been a major global public health issue, ranking third among mortality-causing factors in China [1, 2]. Pancreatic ductal adenocarcinoma (PDAC) is a frequently occurring cancer of the digestive tract, manifesting the worst diagnosis and prognosis among all solid tumors. Its survival rate of 5 years is approximately 10% [3], which is primarily attributed to its metastasis potential. Unfortunately, several basic factors contribute to the high mortality of PDAC. First, because the pancreas is inaccessible in its anatomical position and the tumor generally grows surrounding and invading the blood vessels, only 15–20% of patients can undergo surgery, which is a radical form of therapy [4]. Next, chemotherapy evidently weakens the patients, lowering their tolerance to aggressive therapy and prolonging survival rather than effectively curing them [5]. Finally, PDAC drug resistance is a major problem encountered in improving OS even with the use of radiotherapy or systemic drugs exhibiting high efficacy [6]. Therefore, studying the mechanisms underlying PDAC is of great clinical value and social significance.

The occurrence and progression of pancreatic cancer occur in multiple steps. A total of 90% of patients with PDAC were found to carry KRAS mutations, and 50–80% exhibited inactivated mutations in TP53, CDKN2A, and SMAD4 [7, 8]. SMAD4 is a primary tumor suppressor gene, and its deletion is associated with increased metastasis. The SMAD4 encoded protein includes 552 amino acids with a molecular weight of 60 kDa [9]. Furthermore, as a key member of the transforming growth factor β1 (TGF-β1) pathway, SMAD4 regulates gene expression by activating the signaling pathway as the transcription factor [10, 11]. Canonical TGF-β signaling is correlated with the activation of serine/threonine kinase receptors, thereby facilitating the phosphorylation of receptor-regulated SMADs (R-Smads; SMAD2 and SMAD3). R-Smads and SMAD4 can generate heteromeric complexes, which exhibit nuclear translocation and accumulation [12, 13]. Moreover, R-Smads can modulate the levels of target genes along with cofactors or sequence-specific transcription factors [14]. TGF-β1 is a multifunctional cytokine with several biological functions, including the regulation of cell differentiation, proliferation, apoptosis, and epithelial–mesenchymal transition (EMT) [15, 16]. Therefore, identifying the related genes is crucial for elucidating the pathological mechanism underlying PDAC.

S100A2 was discovered as the EF-hand calcium-binding protein by subtractive hybridization to screen tumor suppressor genes in cancerous versus healthy breast epithelial cells [17, 18]. Several S100 members, such as S100A2, S100A4, and S100A6, are specifically upregulated within aggressive advanced metastatic tumors compared with nonmetastatic and noninvasive tumors. The altered expression of S100A2 is a potential marker for cancer diagnosis and prognosis [19]. Moreover, abnormal expression of S100A2 is associated with diversified cell activities, including proliferation, differentiation, calcium homeostasis, and protein phosphorylation [20, 21]. Ohuchida et al. reported that in pancreatic cancer cases, S100A2 exhibited high expression, leading to dismal prognostic outcomes [22]. Furthermore, Bachet et al. revealed that S100A2 was a predictive factor for pancreatic cancer adjuvant therapy [23]. Studies have revealed that TGF-β1 can directly result in S100A2 gene transcription within epithelial cells by integrating MAPK and p53 signaling pathways [18]. However, the molecular mechanism underlying S100A2 in pancreatic cancer remains to be analyzed.

We found that S100A2 affects cell metastasis and EMT in pancreatic cancer by regulating SMAD4 via the TGF-β/Smad pathway. In this study, the downregulation of S100A2 inhibited PDAC cell metastasis, but its overexpression enhanced cancer metastasis. Thus, S100A2-induced cell metastasis may represent a novel therapeutic target against PDAC.

## Results

### High S100A2 expression in pancreatic cancer cells predicts dismal prognostic outcomes

**Fig. 1:**
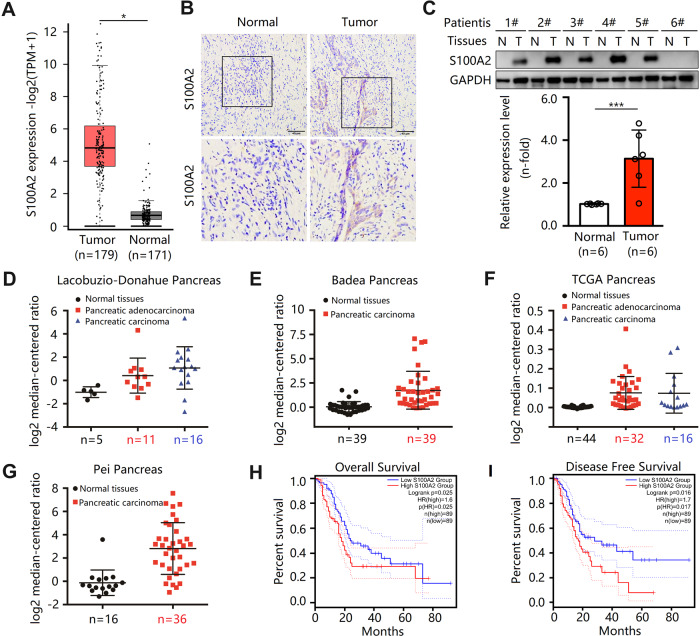
S100A2 expression is upregulated in pancreatic ductal adenocarcinoma and related to poor survival. **A** S100A2 mRNA levels in the noncarcinoma and pancreatic adenocarcinoma tissues. **B** Immunohistochemical representative images of S100A2 in the normal and pancreatic adenocarcinoma tissues. **C** S100A2 protein levels in six sample pairs. **D**–**G** S100A2 mRNA levels in the noncarcinoma and pancreatic adenocarcinoma tissues based on the Oncomine database. **H** Overall survival curves and **I** disease-free survival curves comparing PDAC cases exhibiting high and low levels of S100A2. Data are represented as the mean ± SD. Each assay was performed three times. *P < 0.05; **P < 0.01; ***P < 0.001.

The expression of S100A2 in the PDAC samples increased compared with the noncarcinoma samples (Fig. 1A). To investigate the role of S100A2 in PDAC, we analyzed S100A2 levels in 12 PDAC and 12 matched noncarcinoma samples using immunohistochemical analyses and Western blotting. Based on immunohistochemical analyses, S100A2 levels remarkably increased relative to those in the adjacent nontumor tissues (Fig. 1B). Moreover, the S100A2 protein levels in PDAC samples remarkably increased compared with those in the noncarcinoma samples (Fig. 1C).

Four distinct microarray datasets obtained based on TCGA and Oncomine databases were analyzed; S100A2 expression in most PDAC specimens was significantly higher than that in the normal samples (Table S2). Based on our findings, S100A2 levels in the cancer group significantly increased compared with the normal group, and high S100A2 levels led to poor prognoses and disease-free survival (Fig. 1D–I). Furthermore, the levels of S100A2 were observed under different conditions. We found elevated levels of S100A2 in tumors, correlated with tumor grades, but the promoter methylation level of S100A2 decreased in primary tumors, and the difference in disease was nonsignificant. Moreover, S100A2 expression varied among patients with different skin tones (Fig. S1).

### S100A2 regulation affects pancreatic cancer cell motility in vitro

**Fig. 2:**
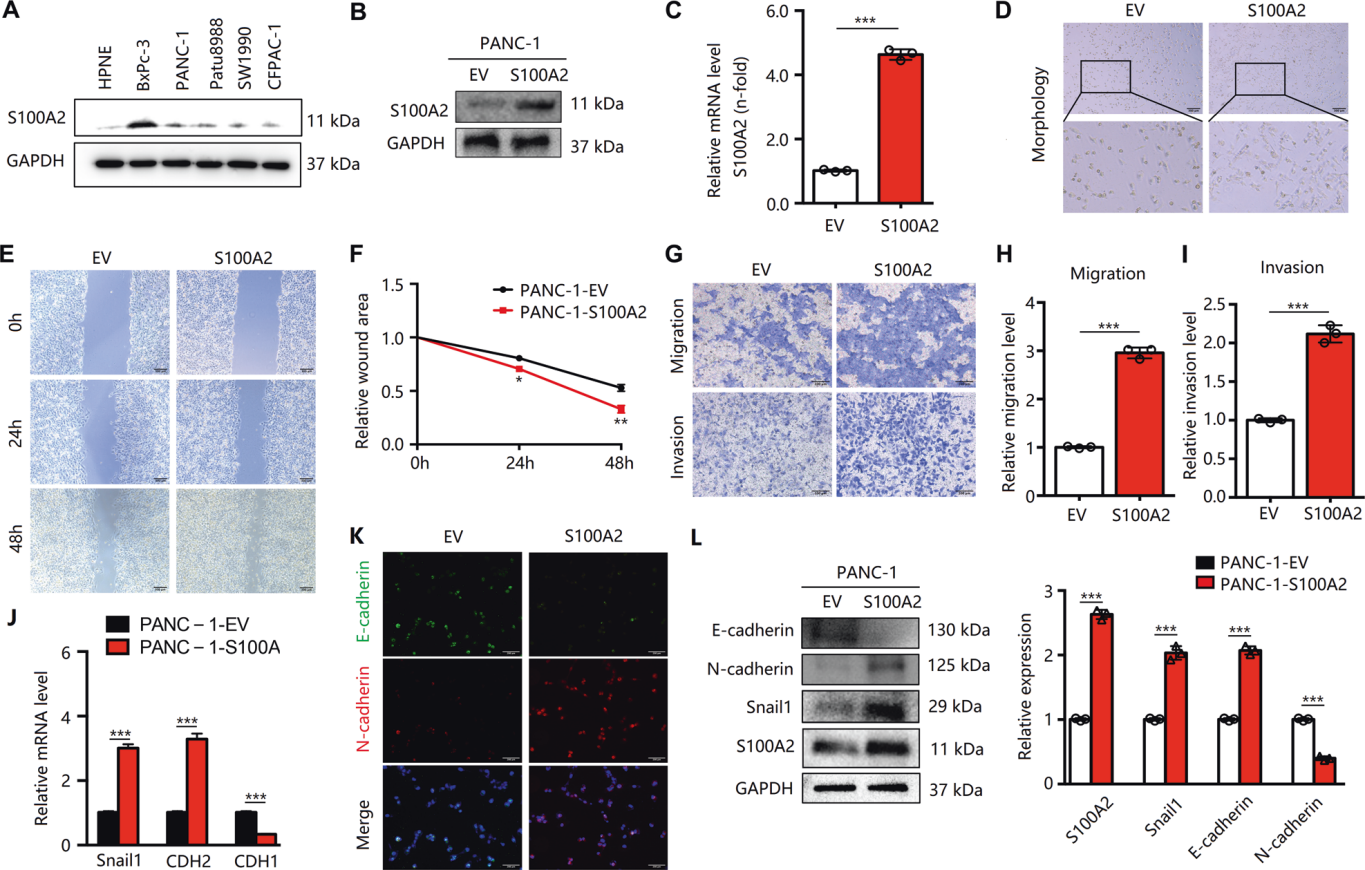
S100A2 regulates pancreatic cancer cell metastasis in vitro. **A** S100A2 levels in the noncarcinoma and pancreatic cancer cells. **B**, **C** Detection of S100A2 in S100A2-overexpressed PANC-1 cells via immunoblotting and qRT-PCR. **D** Comparison of the morphologies of empty vectors and S100A2-overexpressed cells. **E**, **F** According to the scratch-wound assay, S100A2 overexpression remarkably promoted cell invasion. **G**–**I** Based on the Transwell assay, S100A2 overexpression dramatically increased cell invasion and migration. **J**–**L** Overexpression of S100A2 increased EMT, as observed in immunofluorescence, qRT-PCR, and immunoblotting assays. Each assay was performed three times. **P < 0.01; ***P < 0.001.

**Fig. 3:**
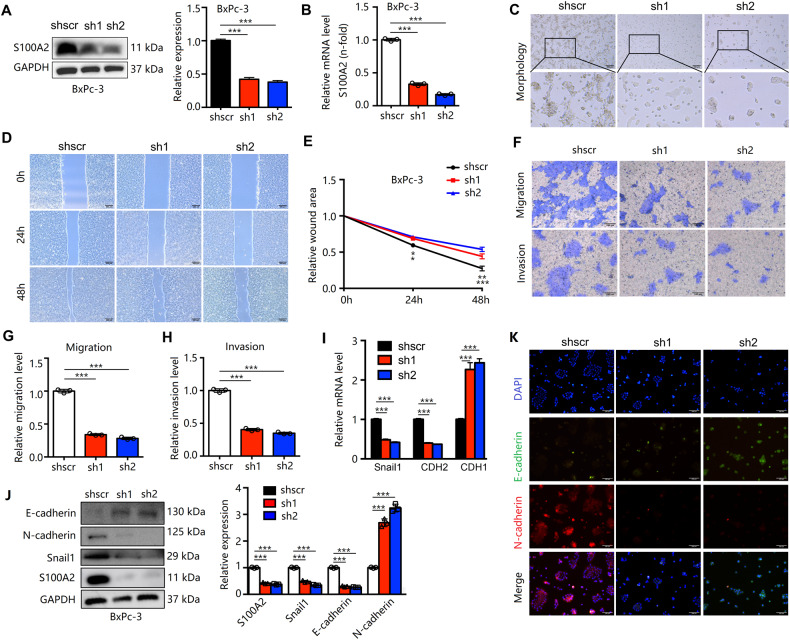
The regulation of S100A2 affects pancreatic cancer cell metastasis. **A**, **B** Detection of S100A2 in S100A2-knockdown BxPC-3 cells via immunoblotting and qRT-PCR. **C** Comparison of the morphologies of empty vector and S100A2-overexpressed cells. **D**, **E** Based on the scratch-wound assay, S100A2 silencing decreased the invasion rate. **F**–**H** According to the Transwell assay, S100A2 knockdown evidently inhibited invasion and migration. **I**–**K** Knockdown of S100A2 inhibited EMT in immunofluorescence, qRT-PCR, and immunoblotting. Each assay was performed three times. **P < 0.01; ***P < 0.001.

To understand the possible effects of S100A2 on PDAC, we analyzed the expression of S100A2 in human pancreatic cancer cells PANC-1, BxPC-3, Patu8988, SW1990, CFPAC-1, and HPNE. S100A2 expression in BxPC-3 was found to remarkably increase compared with other cells and that in PANC-1 cells decreased (Fig. 2A). Therefore, we established the stable overexpression of S100A2 in PANC-1 cells and S100A2 knockdown using the shRNA system in BxPC-3 cells. The efficacy of overexpression was validated using RT-PCR and western blotting (Fig. 2B, C). Based on the results of the scratch-wound and Transwell assays, S100A2 overexpression increased the invasion and migration abilities, and we observed the morphological changes in cells exhibiting overexpression (Fig. 2D–I). The expressions of EMT-associated biomarkers in infected and control cells were determined. S100A2 overexpression significantly increased the mRNA expressions of Snail1 and N-cadherin but significantly reduced the mRNA expression of E-cadherin compared with control cells (Fig. 2J). Furthermore, western blotting and immunofluorescence results revealed similar changes in the levels of these proteins; the level of proteins Snail1 and N-cadherin increased, but that of E-cadherin reduced (Fig. 2K, L). Similarly, we validated the efficacy of S100A2 knockdown using western blotting and RT-PCR (Fig. 3A, B). The scratch-wound and Transwell assays revealed that S100A2 knockdown markedly inhibited metastatic abilities, and morphological changes were observed in the S100A2 knockdown cells (Fig. 3C–H). The EMT-associated biomarkers exhibited expressions contrary to S100A2 overexpression observed in RT-PCR, western blotting, and immunofluorescence assays (Fig. 3I–K).

### S100A2 mitigates the in vivo metastasis and EMT of pancreatic cancer cells

**Fig. 4:**
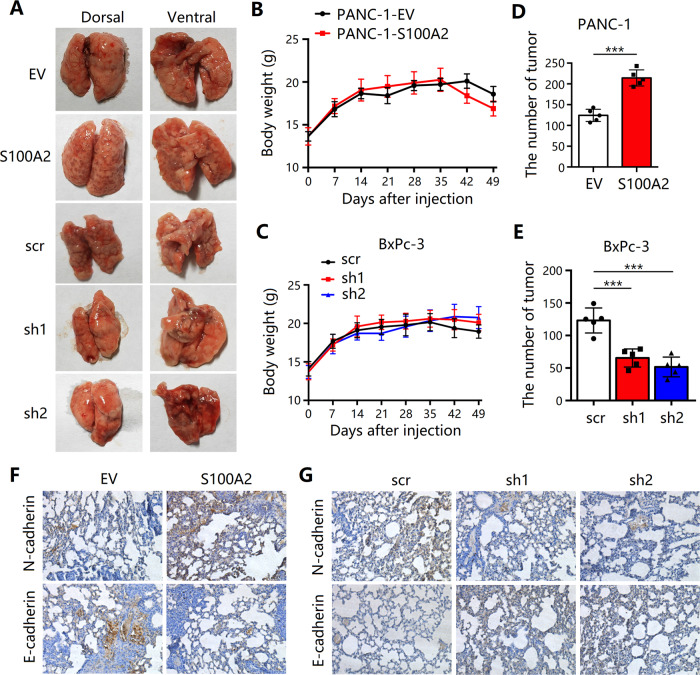
S100A2 mitigates the migration of pancreatic cancer cells via EMT in vivo. **A**–**E** The tumor numbers of the groups with S100A2 overexpression remarkably increased compared with the control group, whereas those of the S100A2-knockdown group reduced. **F**, **G** Immunohistochemical images showing E-cadherin and N-cadherin in the dissected tumor samples from PANC-1 and BxPC-3 cells. Each assay was performed three times. **P < 0.01; ***P < 0.001. *n* = 6 for mice in each group.

To verify our in vitro findings, we analyzed the functions of S100A2 in metastasis by using a xenograft mouse model. As shown in Fig. 4, the group with S100A2 overexpression exhibited markedly elevated tumor numbers compared with the control group, whereas the tumor numbers of the S100A2 knockdown group reduced. The weight of the mice in all groups remained unchanged. Thus, S100A2 mitigated the metastatic ability of PDAC. Furthermore, immunohistochemical staining revealed that the groups with S100A2 overexpression exhibited enhanced N-cadherin expression, whereas the S100A2 knockdown group exhibited reduced expression. E-cadherin expression of the S100A2 knockdown group was found to be elevated but that of the S100A2 overexpression group was reduced. Altogether, S100A2 can promote pancreatic cancer cell metastasis and EMT in vivo and in vitro.

### TGF-β1 induces S100A2 expression to activate the Smad2/3 pathway

**Fig. 5:**
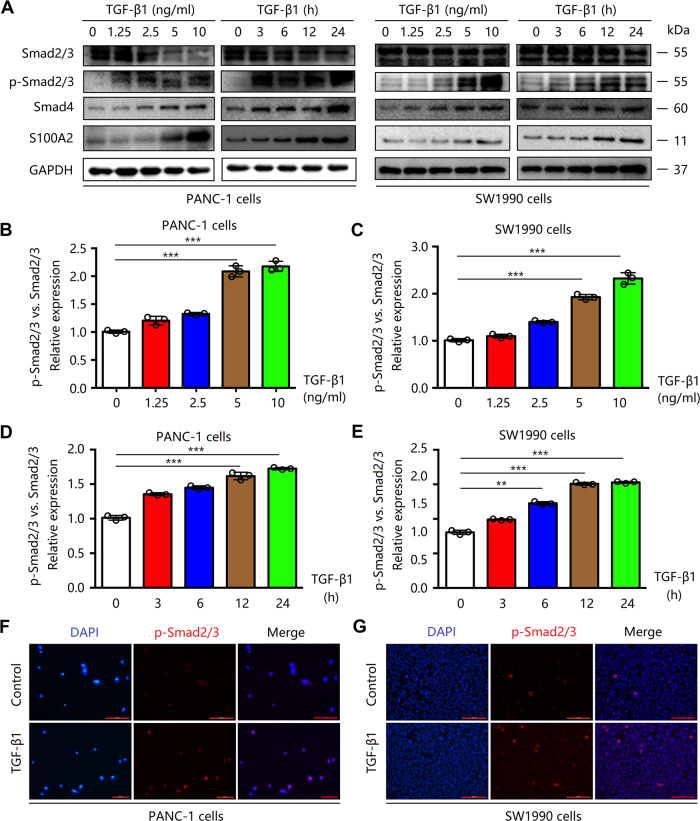
TGF-β1 induced the expression of S100A2 to activate the Smad2/3 pathway. **A**–**E** Immunoblotting assay revealed that TGF-β1 treatment for various time durations (0–24 h) and different concentrations increased the levels of S100A2. **F**, **G** According to immunofluorescence analyses, TGF-β1 promoted S100A2 expression to activate the smad2/3 pathway. Each assay was performed three times. **P < 0.01; ***P < 0.001.

Our previous microarray study revealed that S100A2 is the new downstream target for TGF-β1 within HaCaT and lung epithelial cells [24]. TGF-β1 is correlated with tumor-promoting behaviors such as inducing an EMT that promotes tumor cell migration and invasion. To explore the mechanism underlying the S100A2-mediated regulation of cell invasion and migration in PDAC, we added different concentrations of TGF-β1 to detect S100A2 levels at different time points. The results revealed that exposure to TGF-β1 treatment for different time durations (0–24 h) and different concentrations upregulated the protein levels of S100A2 and SMAD4. Moreover, the expression of psmad23/smad23 was found to increase in PANC-1 and SW1990 cells. As observed via immunofluorescence analyses, TGF-β1 (5 ng/mL) treatment for 24 h increased the level of psmad23 (Fig. 5). The results indicated that TGF-β1 either directly activates the Smad2/3 pathway or indirectly through the expression of S100A2.

### S100A2 affects PDAC cell migration via the regulation of SMAD4

**Fig. 6:**
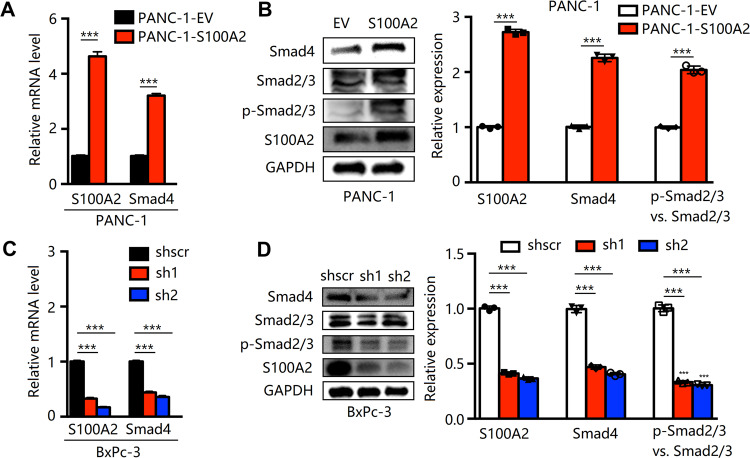
S100A2 positively regulated SMAD4 levels in pancreatic cancer. **A**, **C** SMAD4 gene expressions in PANC-1 and BxPC-3 cells were determined using qRT-PCR. **B**, **D** As revealed by western blotting, S100A2 overexpression increased the levels of Smad2/3/4, and S100A2 knockdown exhibited an opposite effect. Each assay was performed three times. **P < 0.01; ***P < 0.001.

Some studies have revealed that SMAD4 can induce cell metastasis and EMT [12, 25]; thus, to better explore the mechanisms underlying S100A2-mediated regulation of cell metastasis and EMT in PDAC, we studied the expression of SMAD4. As shown in Fig. 6, the SMAD4 levels in cells with S100A2 expression remarkably increased. Western blotting results revealed that S100A2 overexpression promoted an increase in SMAD4 levels, whereas S100A2 knockdown cells exhibited the opposite effect. Moreover, we found that the phosphorylation of smad2/3 increased in S100A2-overexpression cells but decreased in S100A2 knockdown cells.

**Fig. 7:**
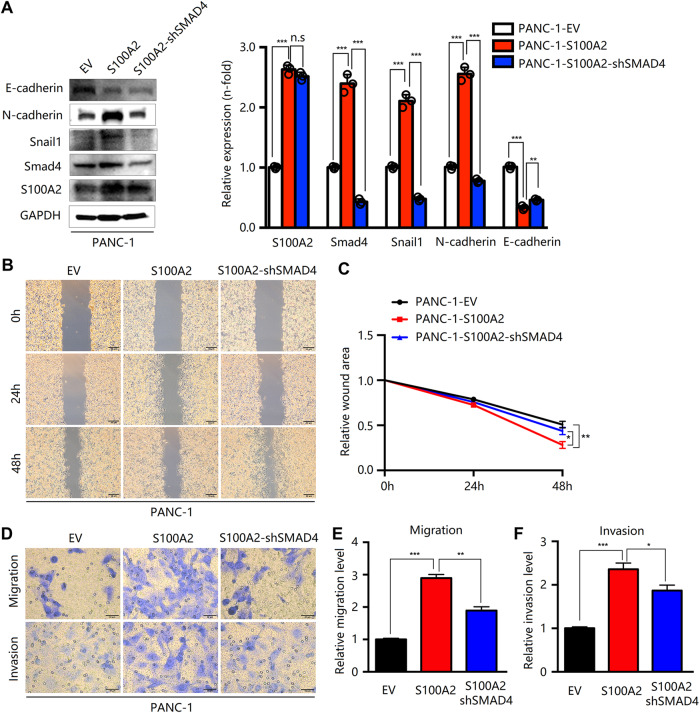
S100A2 affects cell migration via the regulation of SMAD4. **A** Detection of SMAD4 and EMT-related proteins in PANC-1 cells with S100A2 overexpression and SMAD4 knockdown through immunoblotting. **B**, **C** Based on the scratch assay, SMAD4 silencing decreased the invasion rate. **D**–**F** According to the Transwell assay, SMAD4 knockdown inhibited the invasion and migration abilities of cells. Every assay was performed three times. **P < 0.01; ***P < 0.001.

**Fig. 8:**
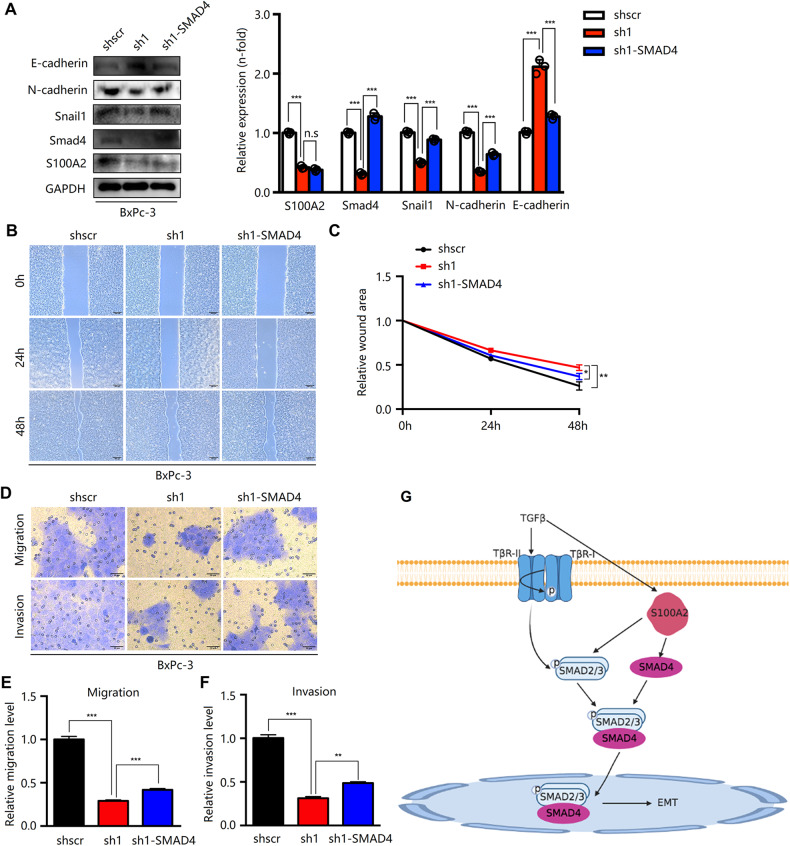
S100A2 regulates EMT and cell metastasis via SMAD4. **A** Detection of SMAD4- and EMT-related proteins in BxPC-3 cells with S100A2 knockdown and SMAD4 overexpression via immunoblotting. **B**, **C** As observed in the scratch-wound assay, SMAD4 overexpression enhanced cell invasion. **D**–**F** According to the Transwell assay, SMAD4 overexpression dramatically increased cell invasion and migration. **G** A proposed model underlying the role of S100A2 in the regulation of EMT and metastasis in PDAC. S100A2 upregulates the expression of SMAD4 through the transforming growth factor (TGF)-β signaling pathway, and the S100A2/SMAD4 axis modulates EMT to accelerate PDAC development. Every assay was performed three times. **P < 0.01; ***P < 0.001.

We subsequently examined whether SMAD4 was a target gene of S100A2 in PDAC cells. Therefore, we established the stable overexpression of SMAD4 in BxPC-3 knockdown S100A2 cells and SMAD4 knockdown by using an shRNA system in PANC-1 S100A2-overexpressing cells. Western blotting revealed that SMAD4 inhibition could effectively eliminate the effects of S100A2 overexpression on EMT (Fig. 7A). According to recent studies, EMT is a migratory cellular program associated with the development and tumor metastasis [26, 27]. Based on scratch-wound and Transwell assays, SMAD4 inhibition decreased the invasion and migration abilities of the cells (Fig. 7B–F). Similarly, SMAD4 overexpression rescued the effects induced by S100A2 knockdown on EMT (Fig. 8A). According to the scratch-wound and Transwell assays, SMAD4 overexpression promoted cell invasion and migration (Fig. 8B–F). Altogether, SMAD4 regulated S100A2 function in EMT and the metastasis of pancreatic cancer cells.

## Discussion

The gene S100A2 is responsible for encoding one 11-kDa acidic protein regulating cell proliferation and cell-cycle [28]. Aberrant S100A2 expression is related to the genesis and development of multiple malignancies [29, 30]. S100A2 is widely recognized as a putative tumor suppressor gene that inhibits cell proliferation. Moreover, its overexpression has been detected in ovarian carcinomas and colorectal cancer [17, 31], indicating that S100A2 critically affects cancer development. However, the effect of S100A2 on pancreatic cancer has rarely been reported [23, 32]. The present study revealed that TGF-β1 activates the Smad2/3 pathway indirectly by the expression of S100A2, which form complexes with SMAD4. Besides, S100A2 promotes cell metastasis and induces SMAD4 expression to regulate the EMT program (Fig. 8G).

The expression of S100A2 is specific, complicated, and different in various tumors [33]. We studied the increase in S100A2 expression by analyzing the Oncomine dataset and pancreatic cancer tissues. Based on our previous results, S100A2 can promote cellular invasion and migration. Wang et al. [34] and Bulk et al. [35] revealed that among early non-small-cell lung cancer cases, S100A2 was the prognostic biomarker or predicting factor for distant metastasis. Zhang et al. [36] reported that the S100A2/KPNA2 cotransport complex modulates the tumor-associated transcription factor NFYA to promote cell metastasis in colorectal cancer. Consistent with these findings, we found that S100A2 overexpression enhanced pancreatic cancer metastasis, whereas S100A2 knockdown resulted in the opposite effect.

For analyzing the mechanism of action of S100A2 in regulating metastasis and EMT, we focused on the TGFβ/Smad signaling pathway-related genes. Many studies have revealed that the signaling pathway promotes tumor cell invasion and metastasis [37]. SMAD4 expression in S100A2 knockdown cells was reduced, whereas that in S100A2-overexpression cells increased. SMAD4 is responsible for encoding a protein essential for the canonical TGFβ pathway. Xia et al. reported that the invasion pattern in human pancreatic cancer organoids was correlated with SMAD4 deficiency, which might predict patient prognosis [38, 39]. In the present study, the inhibition of SMAD4 rescued the S100A2-enhanced metastasis and EMT of pancreatic cancer cells. Therefore, S100A2 was found to enhance metastasis and EMT by increasing the level of SMAD4.

The TGF-β signaling pathway exerts important effects on various cell behaviors, including cancer development and genesis [40]. However, its effect on tumor development is complicated. TGF-β is a tumor suppressor in healthy and dysplastic cells; however, it acts as the tumor promoter in advanced cancers [41]. In this study, TGF-β1 significantly upregulated S100A2 expression. TGF-β1 induced S100A2 expression to activate the smad2/3/4 pathway. Therefore, S100A2 may modulate SMAD4 levels via the TGF-β /Smad pathway.

This study provides evidence for the involvement of TGF-β1 in S100A2-mediated cell metastasis and EMT during the development of pancreatic cancer. Moreover, we found that S100A2 is a novel regulatory factor for pancreatic cancer cell metastasis, with SMAD4 being a potential downstream target, and together they form the S100A2/SMAD4 axis to promote cellular invasion and migration.

## Materials and methods

### Cell culture and treatment

Human PDAC cell lines (PANC-1, BxPC-3, Patu8988, SW1990, and CFPAC-1) were obtained from the Chinese Academy of Sciences Cell Bank (Shanghai, China) and cultured in RPMI-1640 medium, whereas HEK293T and human pancreatic normal cells (HPNE) were cultured in Dulbecco’s modified Eagle medium (DMEM) containing 10% fetal bovine serum (FBS; Thermo Fisher Biotechnology, Shanghai, China) under conditions of 5% CO_2_ and 37 °C.

For TGF-β1 analysis, cells were cultured and serum starved for 24 h before treatment with TGF-β1 (R&D Systems) at a concentration of 0, 1.25, 2.5, 5, or 10 ng/mL for different time points (0, 3, 6, 12, 24 h). Finally, cells were treated with TGF-β1 (5 ng/mL) for 24 h.

To obtain overexpression constructs, we amplified the entire coding sequence of S100A2 or SMAD4 and then inserted it in pcDNA3.1 (Invitrogen, CA, USA). For S100A2 and SMAD4 knockdown, S100A2-specific and SMAD4-specific shRNAs were cloned into vector pLKO.1. The following pairs of shRNA were used: S100A2, 5′-CCGGCGACAAGTTCAAGCTGAGTAACTCGAGTTACTCAGCTTGAACTTGTCGTTTTTG-3′-F, 5′ AATTCAAAAACGACAAGTTCAAGCTGAGTAACTCGAGTTACTCAGCTTGAACTTGTCG-3′-R; SMAD4, 5′CCGGGTACTTCATACCATGCCGATTCTCGAGAATCGGCATGGTATGAAGTACTTTTTG-3′-F, 5′- AATTCAAAAAGCAGACAGAAACTGGATTAAACTCGAGTTTAATCCAGTTTCTGTCTGC-3′-R. Transfection was conducted using lipofectamine 3000 reagents (Invitrogen, CA, USA) according to specific instructions. Stable clones of overexpressed and knockdown cells were selected with G418 and puromycin, respectively.

### Xenograft tumor model

This study included 5-week-old male BALB/c nude mice from the Vital River Laboratory Animal Technology (Beijing, China). The mice were housed under specific pathogen-free conditions at the Laboratory Animal Center of Wenzhou Medical University (Wenzhou, China). To investigate the effect of S100A2 on metastatic tumors in vivo, 1 × 10^6^ PANC-1 cells (transfected with S100A2 or a vector) and 5 × 10^6^ BxPC-3 cells (with/without S100A2 knockdown) were administered to each mouse via subcutaneous injections through the tail vein. Considering excess tumor burdens, each mouse was humanely sacrificed after 7 weeks. The tumor sections were immunohistochemically stained for E-cadherin and N-cadherin.

### Oncomine/the cancer genome atlas dataset analyses

Relative S100A2 mRNA expression in pancreatic cancer was obtained based on the Cancer Genome Atlas (TCGA) database (https://www.TCGA.org) and Oncomine Cancer Microarray database (https://www.oncomine.org), both of which can be freely accessed online. Data were later processed based on previous protocols.

### Ethics statement

A total of 12 paired tissue samples was intraoperatively collected at the First Affiliated Hospital of Wenzhou Medical University, and we analyzed the level of S100A2 protein. We obtained approval from the Ethics Committee of the First Affiliated Hospital of Wenzhou Medical University (No. 2019-011) and conducted the experiments following the Declaration of Helsinki. Each participant provided informed consent prior to participation. The animal study was performed in compliance with the Guide for the Care and Use of Laboratory Animals. Our study protocols were approved by the Institutional Animal Care and Use Committee of Wenzhou Medical University.

### Western blotting

Radioimmunoprecipitation assay buffer containing phosphatase inhibitors (Beyotime, China) was used to lyse tissues and cells on ice. The cells were then subjected to 20-min centrifugation at 10,000 × *g* and 4 °C to obtain supernatants. The Bradford assay kit (Beyotime, China) was subsequently used to analyze the total protein content. Protein samples were loaded, electrophoresed, and transferred onto a hydrophilic polyvinylidene fluoride membrane (Millipore, Billeria). The membranes were then incubated within 5% defatted milk under ambient temperature for 2 h and further incubated using specific antibodies at 4 °C overnight. Subsequently, the proteins were incubated for another 1 h with secondary antibodies under ambient temperature. The enhanced chemiluminescence detection kit (Millipore, Billeria) was used to visualize bands, and Image J software was used for analyses. Primary antibodies used in this study included: S100A2 (Ab109494), N-cadherin (Ab76011), E-cadherin (BS72286), Snail-1 (I3099-I-AP), p-Smad2/3 (AP0326), Smad2/3 (AF6367), SMAD4 (SC7966), and GAPDH (AC033).

### Quantitative real-time polymerase chain reaction

RNAiso reagent (Takara Biomedical Technology, Beijing, China) was used for extracting total RNA, whereas the PrimeScript^TM^ RT reagent ((TaKaRa, RR037A, Tokyo, Japan) was used to prepare cDNA samples based on specific protocols. Quantitative real-time polymerase chain reaction (qRT-PCR) was performed using a 7500 fast System with a SYBR Premix Ex Taq™ II kit (Takara, Dalian, China). Subsequently, mRNA levels were calculated based on the ΔΔCT, and GAPDH was considered the reference. Table S1 lists the primer sequences.

### Immunohistochemistry and immunofluorescence

For immunohistochemical analyses, the tissues were deparaffinized, rehydrated, and incubated for 10 min with 3% H_2_O_2_ to quench endogenous peroxidase. Subsequently, the tissue sections were rinsed three times using phosphate-buffered saline and then blocked for 1 h with 5% BSA at 37 °C. They were later incubated with primary antibodies directed against S100A2 (1:200, Ab109494), N-cadherin (1:200, Ab76011), or E-cadherin (1:200, BS72286) overnight at 4 °C. Subsequently, horseradish peroxidase-labeled secondary antibodies (1:500, Cell Signaling Technology, Shanghai, China) were added, and the mixture was subjected to 1-h incubation. All sections were examined under a light microscope (Nikon, Japan). For immunofluorescence analyses, the staining protocol was similar to that used for the immunohistochemical analyses, and the only difference was treatment with fluorescent secondary antibodies The samples were semi-quantitatively or quantitatively evaluated by two independent researchers who were blinded to group allocation.

### Scratch-wound assay

Pancreatic cancer cells (1 × 10^6^ cells) were inoculated in 6-well plates with culture medium. Later, a 200-μL pipette tip was used to create scratches after the cells attained 90% confluence. After discarding the medium, the cells were washed and further cultivated in serum-free DMEM. Furthermore, the scratch width was monitored at 24 and 48 h and then photographed.

### Transwell assay

Pancreatic cells were resuspended at 5 × 10^4^ cells per 80 mL of serum-free medium and then introduced into the top Transwell chamber with/without matrigel (1:10) for determining cell migration and invasion. Subsequently, a medium supplemented with 10% FBS was introduced in the bottom chamber, and the cells were incubated for another 48 h. Next, cells migrating to the upper membrane surface were fixed using methanol for 15 min before crystal violet staining. The light microscope was used to calculate the number of invading or migrating cells.

### Statistical analyses

Statistical analysis was performed using SPSS16.0. Mean data between two groups were compared with a two-sided Student’s *t*-test, whereas one-way ANOVA with Bonferroni’s post-test was used to compare multiple groups. The data were expressed as the mean ± standard error of the mean. *P* < 0.05 was set as the threshold of statistical significance.

### Supplementary information

Supplementary Tables

Supplementary Figure 1

Supplementary Material-western blots

## Data Availability

All data generated and analyzed during this study are included in this article and supplementary file.
