# Supplementary material for: S100A2 induces epithelial–mesenchymal transition and metastasis in pancreatic cancer by coordinating transforming growth factor β signaling in SMAD4-dependent manner
Source: Cell Death Discov. 2023 Sep 27;9:356. doi: 10.1038/s41420-023-01661-1 (PMC10533899; doi:10.1038/s41420-023-01661-1)
Supplement: Supplementary file 1 — Supplementary Tables [file 41420_2023_1661_MOESM1_ESM.docx]

**Table S1: Primers for real-time PCR**

| Gene | Forward | Reverse |
| --- | --- | --- |
| S100A2 | CCAAGAGGGCGACAAGTT | TGATGAGTGCCAGGAAAA |
| CDH1 | GCCTCCTGAAAAGAGAGTGGAG | TGGCAGTGTCTCTCCAAATCCG |
| CDH2 | CCTCCAGAGTTTACTGCCATGAC | GTAGGATCTCCGCCACTGATTC |
| Snail1 | AAGATGCACATCCGAAGCCA | CATTCGGGAGAAGGTCCGAG |
| SMAD4 | CCATCCAGCATCCACCAAGT | TGTCGATGACACTGACGCAA |
| Actin | GTCATTCCAAATATGAGATGCGT | GCTATCACCTCCCCTGTGTG |
| GAPDH | ACCACAGTCCATGCCATCAC | TCCACCACCCTGTTGCTGTA |

**Table S2: Oncomine dataset analysis**

| **Datasets (sample size)** | **Comparison groups** | **Fold Change** | ***P* value** |
| --- | --- | --- | --- |
| Lacobuzio-Donahue Pancreas (32) | Pancreatic Adenocarcinoma vs. Normal  Pancreatic Carcinoma vs. Normal | 2.681  3.427 | 3.24E-7  6.11E-8 |
| Badea Pancreas (78) | Pancreatic Carcinoma vs. Normal | 3.277 | 5.32E-8 |
| TCGA Pancreas (92) | Pancreatic Adenocarcinoma vs. Normal | 2.325 | 2.10E-7 |
|  | Pancreatic Carcinoma vs. Normal | 2.226 | 1.66E-7 |
| Pei Pancreas (52) | Pancreatic Carcinoma vs. Normal | 5.782 | 4.78E-13 |
