# Supplementary material for: S100A2 induces epithelial–mesenchymal transition and metastasis in pancreatic cancer by coordinating transforming growth factor β signaling in SMAD4-dependent manner
Source: Cell Death Discov. 2023 Sep 27;9:356. doi: 10.1038/s41420-023-01661-1 (PMC10533899; doi:10.1038/s41420-023-01661-1)
Supplement: Supplementary file 2 — Supplementary Figure 1 [file 41420_2023_1661_MOESM2_ESM.docx]

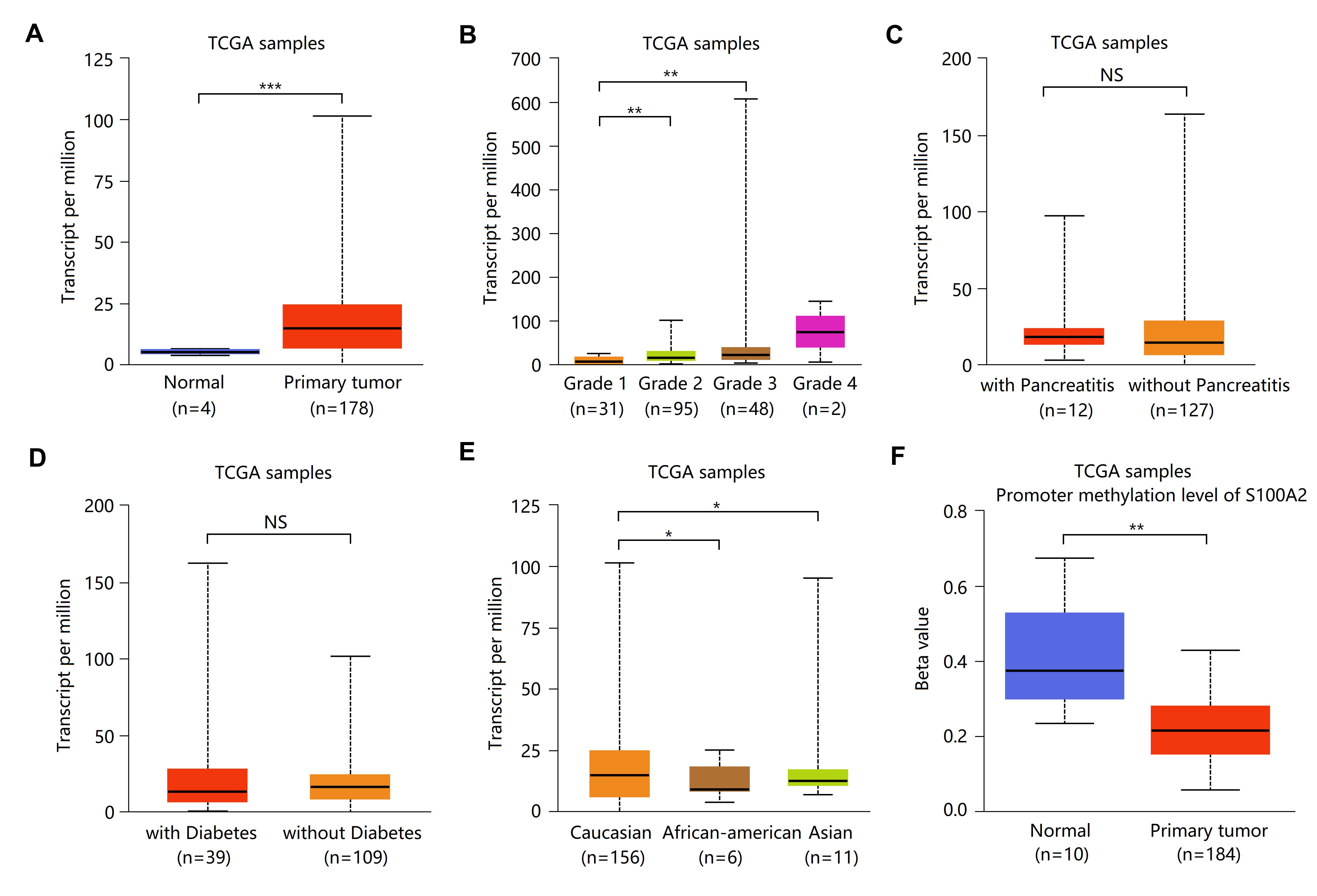


**Figure S1. The expression of S100A2 in different situations.** (A-F) Comparison of S100A2 mRNA expression in different situations from TCGA dataset. Each analysis was performed by triplicate.*P < 0.05; **P < 0.01; ***P < 0.001.
