# Supplementary figures and images for: S100A2 induces epithelial–mesenchymal transition and metastasis in pancreatic cancer by coordinating transforming growth factor β signaling in SMAD4-dependent manner
Source: Cell Death Discov. 2023 Sep 27;9:356. doi: 10.1038/s41420-023-01661-1 (PMC10533899; doi:10.1038/s41420-023-01661-1)

Figure 1

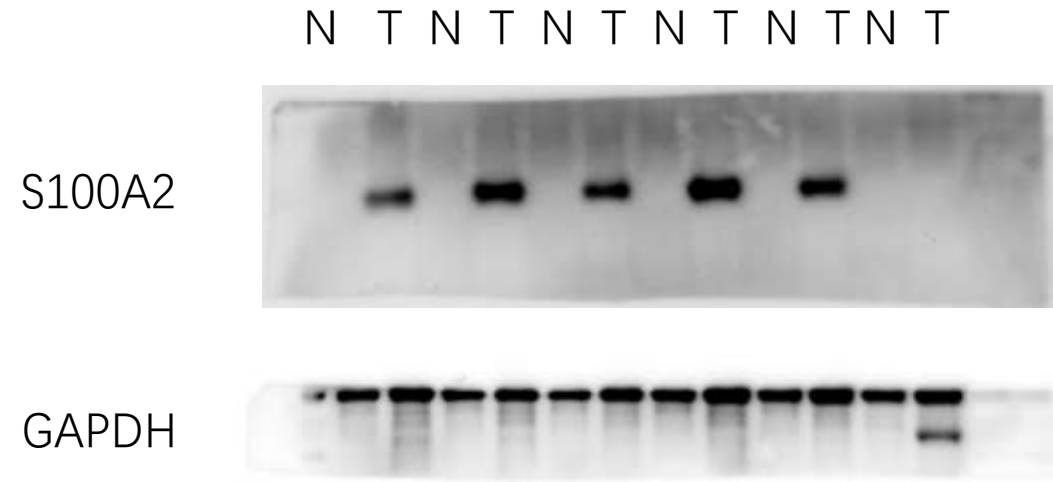

Figure 2

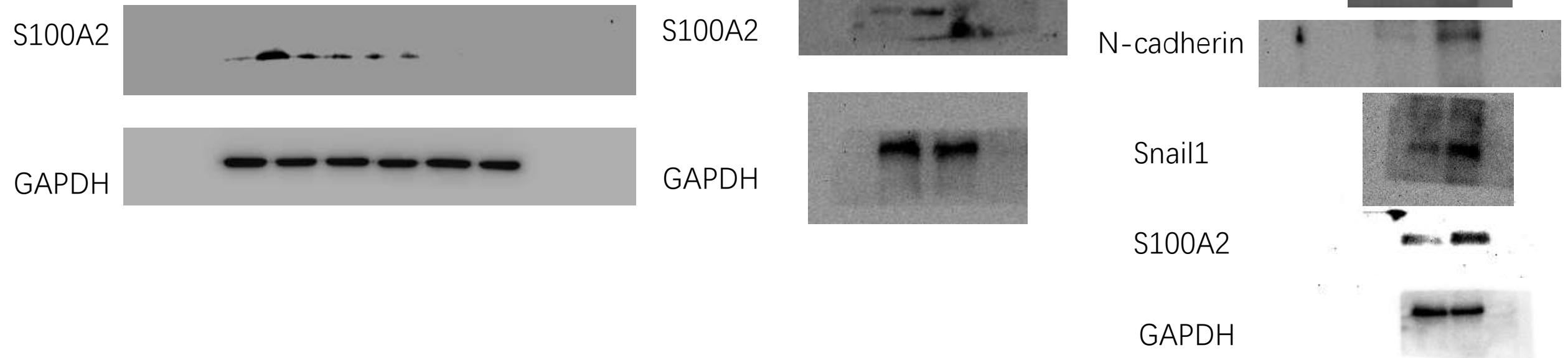

Figure 3

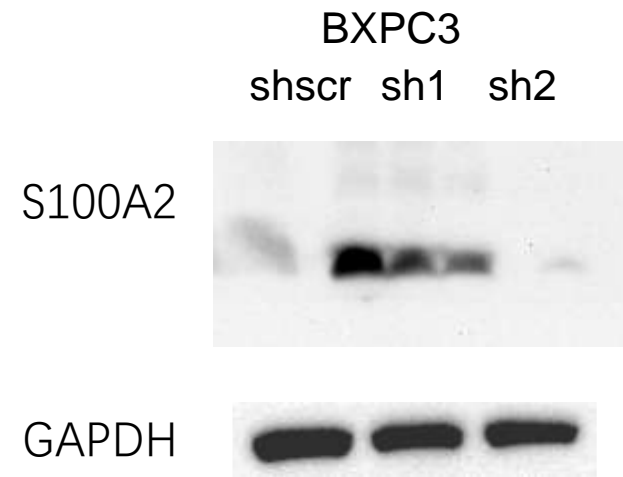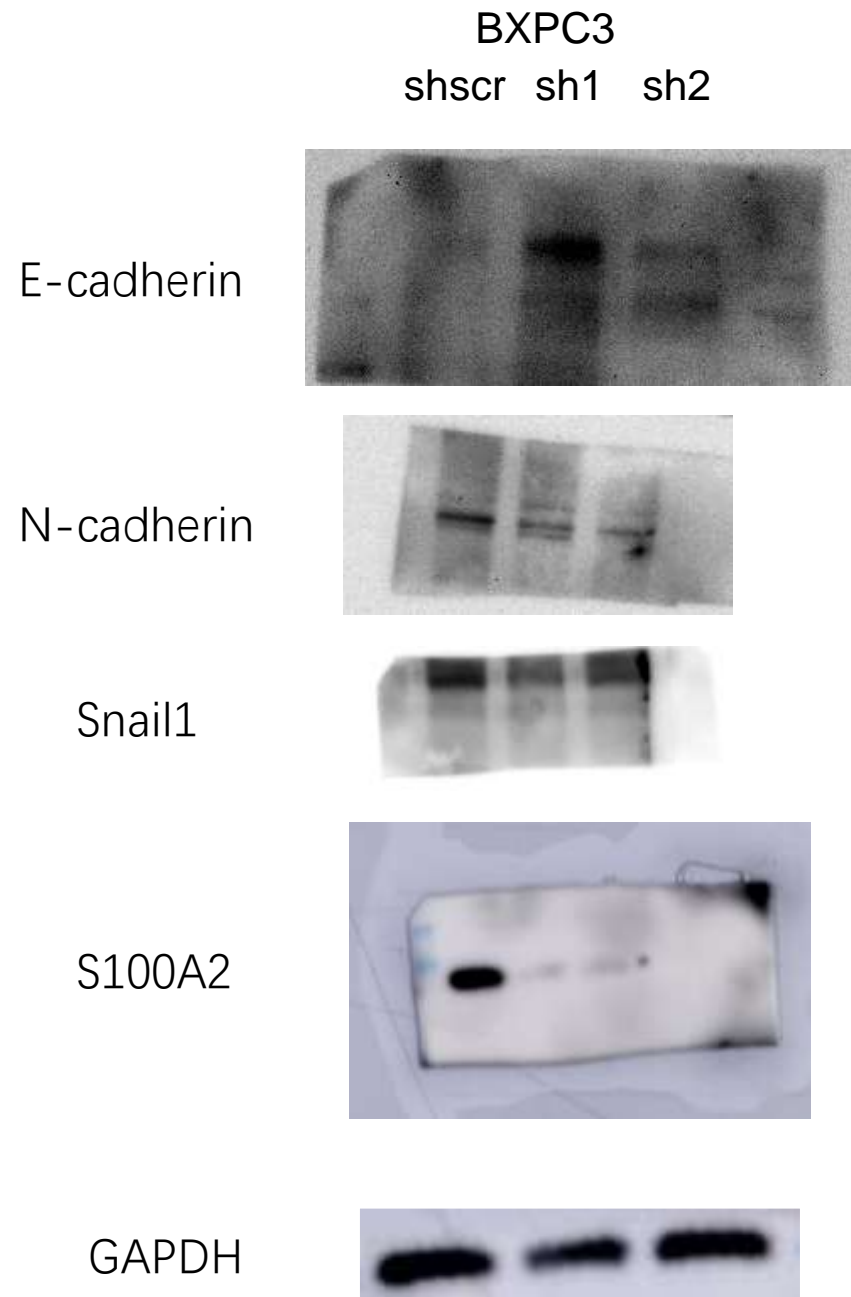

Figure 5

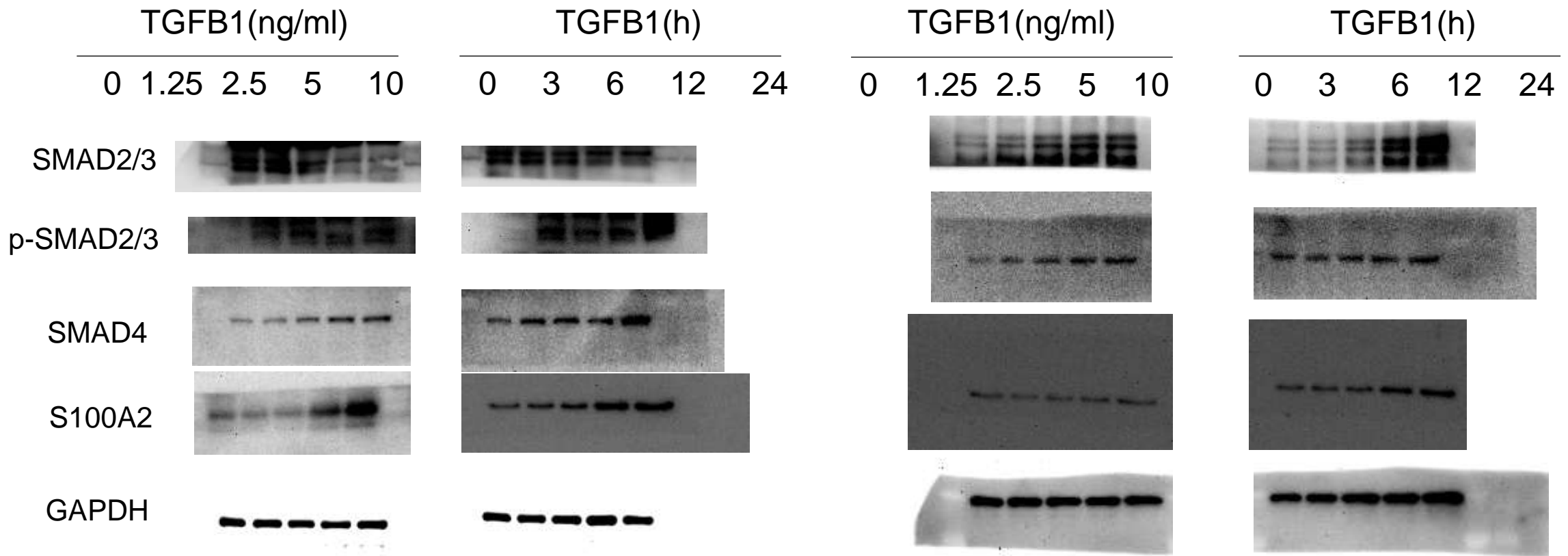

Figure 6

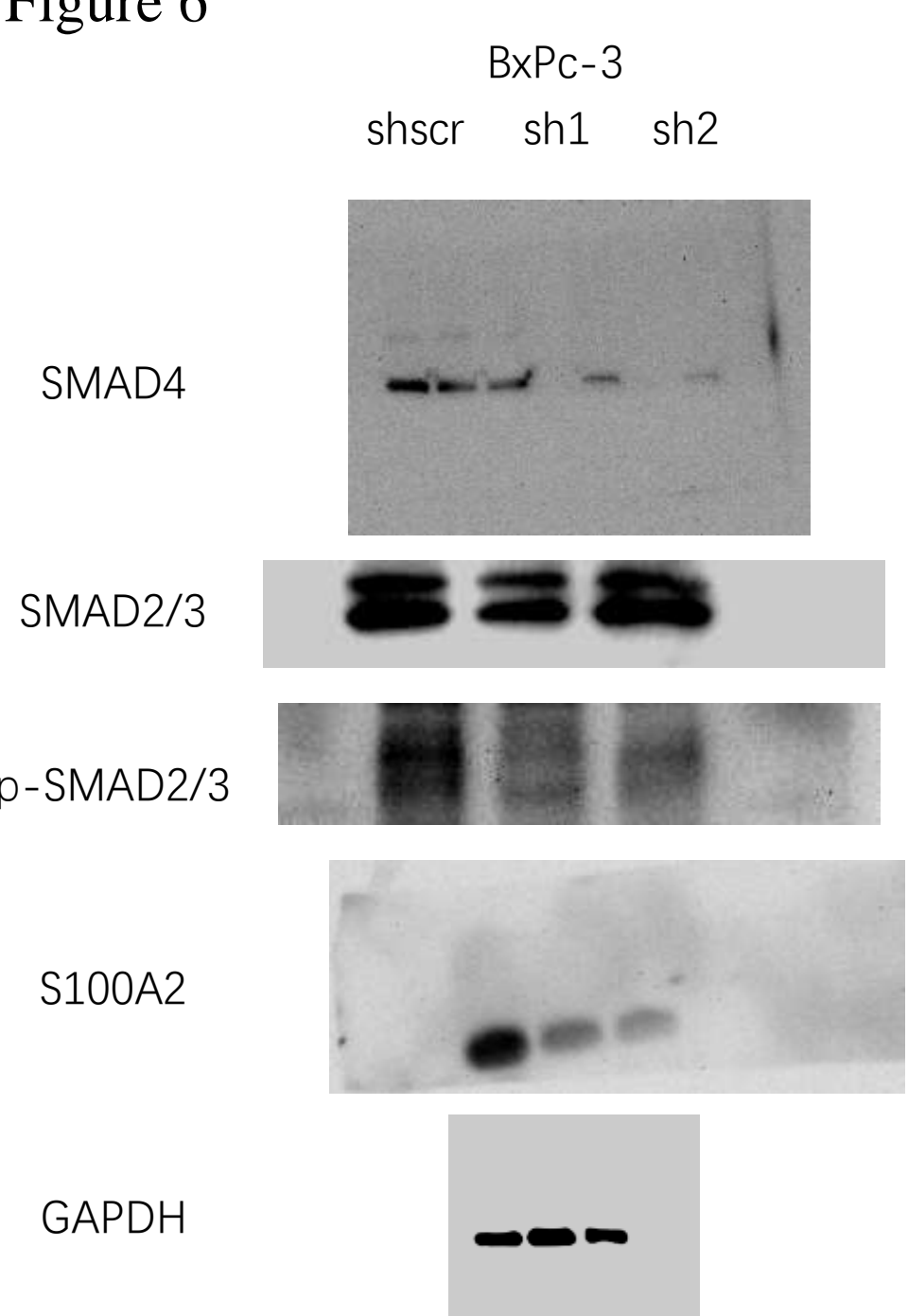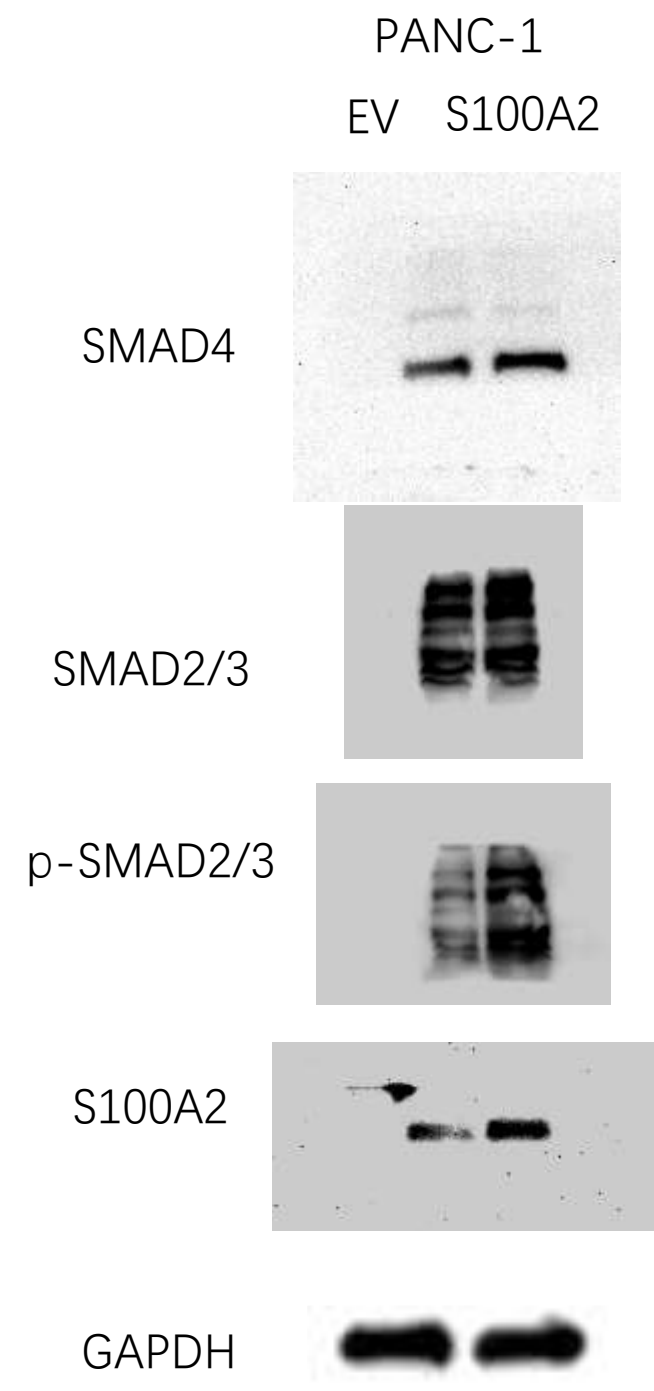

Figure 7

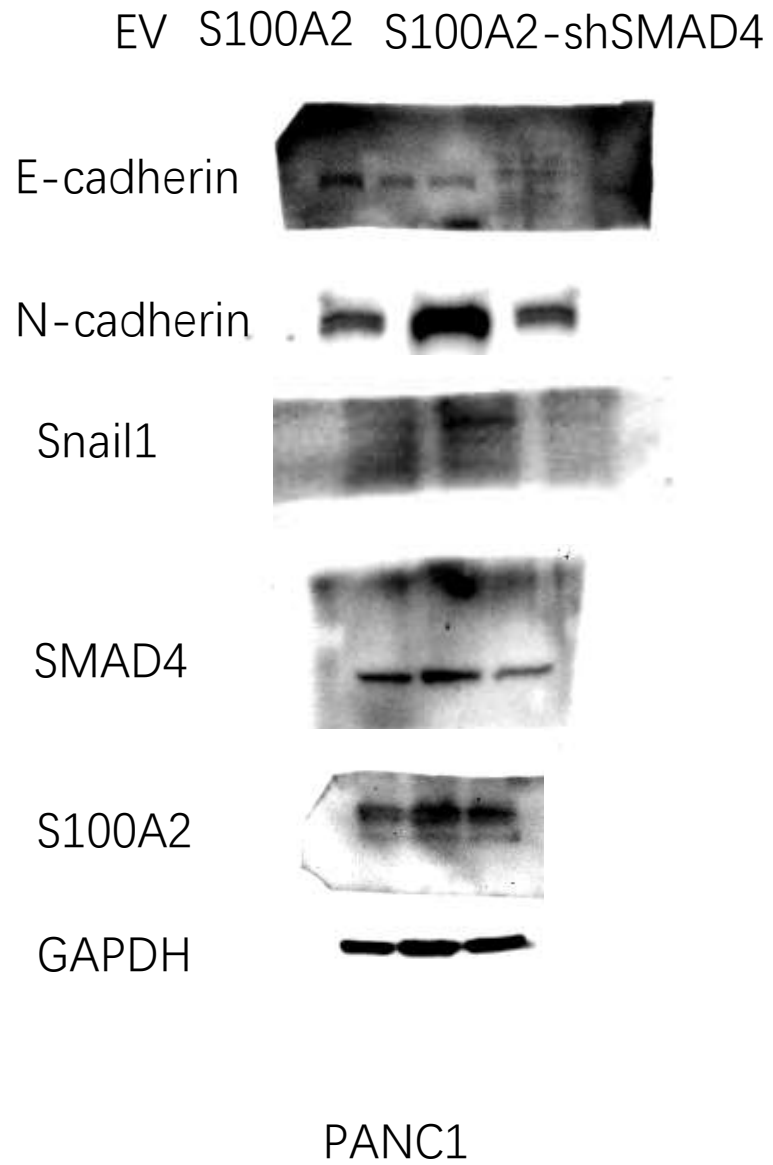

Figure 8

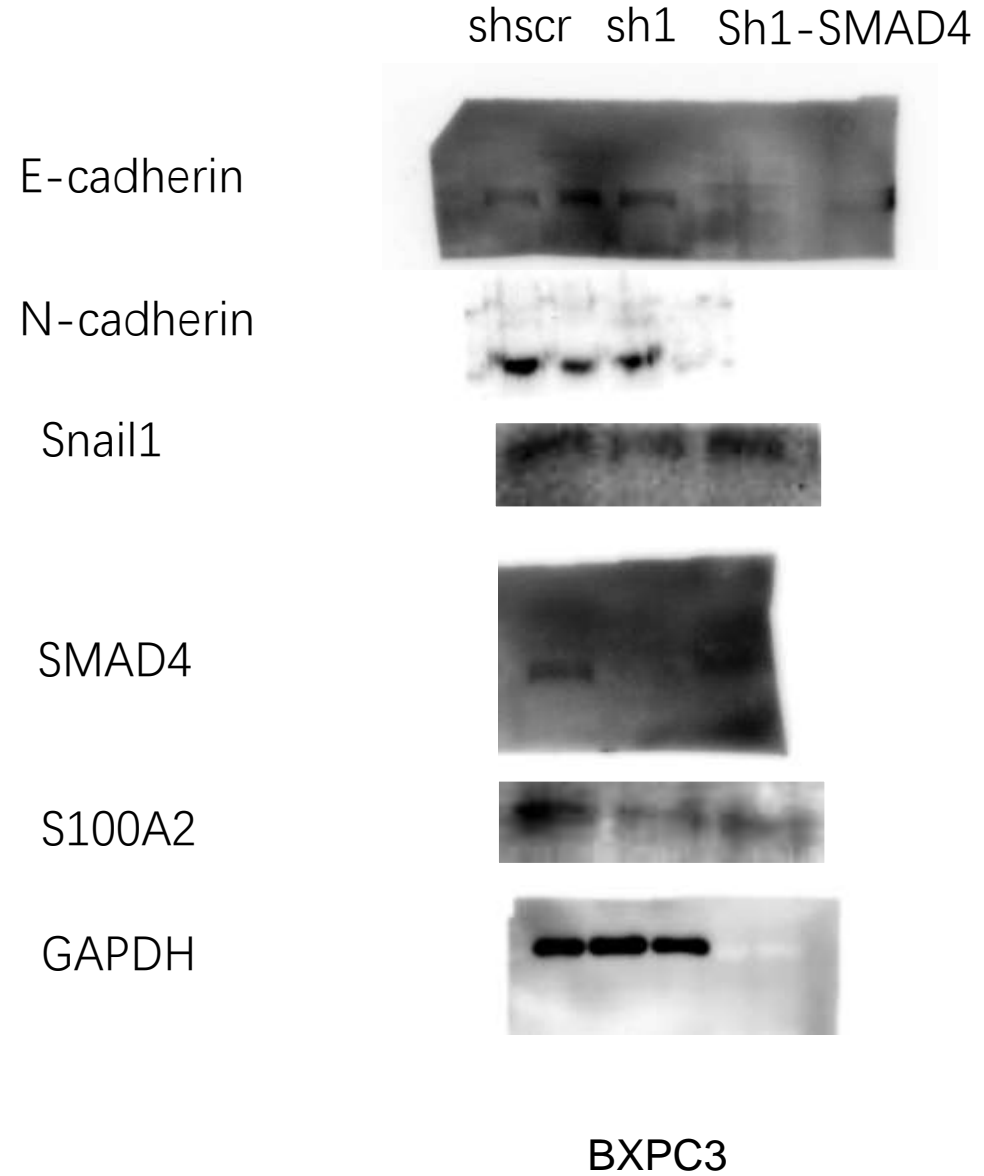

Supplement: Supplementary file 3 — Supplementary Material-western blots [file 41420_2023_1661_MOESM3_ESM.pdf]
